# 早期非小细胞肺癌个体化手术策略与术中冰冻病理的指导

**DOI:** 10.3779/j.issn.1009-3419.2016.06.13

**Published:** 2016-06-20

**Authors:** 彬 胡, 强 李

**Affiliations:** 610000 成都，四川省肿瘤医院胸外科 Department of Thoracic Surgery, Sichuan Cancer Hospital, Chengdu 610000, China

**Keywords:** 肺肿瘤, 亚肺叶切除术, 术中冰冻病理, Lung neoplasms, Sub-lobectomy, Intraoperative frozen section

## Abstract

早期非小细胞肺癌尤其是电子计算机断层扫描（computed tomography, CT）筛查出的微小的在影像学表现为磨玻璃密度样（ground-glass opacity, GGO）的肺癌在亚洲人群中逐渐增多。临床回顾性数据表明对此类患者施行亚肺叶切除术疗效可能并不次于肺叶切除，而且保留了更多的肺功能。研究证明除仔细地评估术前影像表现外，术中冰冻病理诊断为原位腺癌或微浸润肺腺癌可能更适合做亚肺叶切除。更精准的个体化手术策略应基于术中快速冰冻病理诊断。

## Abstract (en)

Early stage non-small cell lung cancer patients, especially those who presented small GGO on image, are increasing in Asia population. Retrospective clinical data indicated that the efficacy of sub-lobectomy for these patients may be non-inferior to that of lobectomy. Meanwhile, sub-lobectomy could preserve more pulmonary function for the patients. Researches have demonstrated that careful evaluation for the image manifestation is essential before sub-lobectomy and those who diagnosed as adenocarcinoma in situ or minimally invasive adenocarcinoma may be more appropriate for this procedure. Precise diagnosis of Intraoperative frozen section is an effective method toward personalization of surgical care for early-stage non-smallcell lung cancer.

美国肺癌研究组（Lung Cancer Study Group, LCSG）于1995年报道了比较肺叶切除与亚肺叶切除治疗非小细胞肺癌（non-small cell lung cancer, NSCLC）的前瞻性多中心随机对照研究，并发症、死亡率和术后肺功能等方面亚肺叶切除术与肺叶切除术相比无统计学差异，复发率亚肺叶切除术是肺叶切除术的3倍，肺叶切除术因此被确立为治疗T1N0M0，Ⅰa期NSCLC的标准术式^[1]^。该研究病例入组时间是1982年-1988年，当时没有应用电子计算机断层扫描（computed tomography, CT）术前做X线检查评估病情，也没有正电子发射计算机断层显像（positron emission tomography, PET）作为术前分期检查；入组太多的楔形切除病例（32.8%），解剖性肺段切除的病例是否具有优势未得到分析；肿瘤直径 > 2 cm的病例较多，影响复发率的准确性等方面存在明显的局限性。近年来，随着低剂量螺旋计算机断层扫描在肺癌高危人群筛查中的应用，越来越多的早期肺癌被发现^[2]^。目前许多研究致力于早期周围型小病灶肺癌亚肺叶切除术能否取代标准术式肺叶切除术，以及如何制定个体化治疗策略。

## 1 亚肺叶切除术的手术方式

亚肺叶切除术包括解剖性肺段切除术和楔形切除术。研究^[3-7]^认为肺段切除术优于楔形切除术，Ⅰ期NSCLC解剖性肺段切除术在复发率及生存率方面具有优势。肺段切除术肿瘤切缘距离更合理，更有可能彻底地切除段及段间引流的淋巴管，此淋巴管被认为是肿瘤复发的重要来源^[8]^。El-Sherif等^[3]^回顾性了81例NSCLC亚肺叶切除术的临床资料，发现行肺楔形切除术的肿瘤距离切缘超过1 cm仅39%（21/55），而肺段切除术达到73%（19/26），楔形切除术的局部复发率高于肺段切除术。Schuchert等^[9]^回顾了182例Ⅰ期NSCLC患者行解剖性肺段切除术的临床资料，平均肿瘤距离切缘1.82 cm，32例在术后出现复发转移（平均14.3个月），其中14例为局部复发，18例为远处转移。在复发转移的患者中，86%的患者切缘距离≤2 cm，切缘距离与肿瘤直径的比值大于1的患者复发率要远低于比值小于1的患者。Raviaro等^[10]^于1993年首次报道胸腔镜肺段切除术。Ⅰa期NSCLC胸腔镜下解剖性肺段切除术与肺叶切除术相比，淋巴结切除、局部复发率、生存率无统计学差异，同时胸腔镜手术微创的优势对患者更为有利^[9]^。

## 2 亚肺叶切除术的适应征

目前研究总结的亚肺叶切除术适应征主要包括：高龄、心肺功能差以及患有不能耐受肺叶切除术的合并症；临床诊断为周围型Ⅰa期NSCLC，肿瘤直径≤2 cm；影像诊断为磨玻璃密度样（ground-glass opacity, GGO）的结节；病理诊断为原位腺癌（adenocarcinoma *in situ*, AIS）或微浸润肺腺癌（minimally invasive adenocarcinoma, MIA）。

### 2.1 高龄、心肺功能差的NSCLC患者肺部手术的风险相对较高

多项研究^[11-15]^表明与肺叶切除术相比，亚肺叶切除术治疗心肺功能差的高龄早期NSCLC患者，肺功能损伤小，手术风险明显降低，5年生存率及局部复发率等方面无统计学差异。

### 2.2 肿瘤大小

肿瘤直径的大小与患者的预后密切相关，多项回顾性临床研究已经证实亚肺叶切除术用于≤2 cm和 > 2 cm的肿瘤会产生不同的预后。2005年Okada^[16]^回顾了1, 272例早期患者NSCLC的临床资料，肺段切除术和肺叶切除术在≤2 cm的肿瘤中5年DFS没有统计学差异，分别为84.6%和87.4%。Carr等^[17]^回顾了429例Ⅰa期NSCLC患者的临床资料，发现肿瘤直径≤2 cm的患者5年无病生存（disease free survival, DFS）为86%，而 > 2 cm的患者为75%（*P*=0.027）。Nomori等^[18]^近期回顾了179例周围型cT1N0M0 NSCLC肺段切除术，术中快速病理来确认切缘距离肿瘤至少2 cm以上，肿瘤直径≤2 cm和2.1 cm-3.0 cm的患者5年生存率分别为94%和81%（*P*=0.006）。目前一些针对≤2 cm的亚肺叶切除术对比肺叶切除术的Ⅲ期随机临床对照研究正在进行中。

### 2.3 GGO病变及特殊病理类型

随着影像学技术的进步，越来越多的GGO病变被胸部薄层CT发现。GGO病变通常倾向于AIS或MIA，手术切除临床获益大^[2]^。目前研究表明这一类患者亚肺叶切除术有可能替代作为标准术式的肺叶切除。Tsutani等^[19]^分析了239例GGO临床Ⅰa期肺腺癌患者临床资料，其中有90例肺叶切除术、56例肺段切除术、93例肺楔形切除术，术后3年DFS分别为96.4%、96.1%和98.7%，三者无统计学差异（*P*=0.44）。Sugi等^[20]^报道肿瘤直径 < 1.5 cm、GGO成分占比 > 75%的周围型肺癌，胸腔镜肺楔型切除手术的5年DFS可达到100%，而肿瘤直径在1.5 cm-2 cm之间者，行胸腔镜解剖性肺段切除联合纵隔淋巴结采样术的5年DFS为90.5%。近期Nakao等^[21]^一项研究发现，26例 < 2 cm的外周型NSCLC患者行亚肺叶切除术后，5年的复发率为0。外周小病灶非黏液型BAC患者亚肺叶切除术DFS可达到100%^[22]^。

## 3 亚肺叶切除术式个体化选择与术中冰冻病理指导

亚肺叶切除术适用于高龄、心肺功能差以及患有不能耐受肺叶切除术的患者，这类适应征属于风险较高的患者手术设计的被动选择。手术风险相对低的患者根据肿瘤直径大小，影像诊断GGO及其实变比例，组织病理学类型为原位或微浸润肺腺癌，肿瘤离切缘距离，淋巴结转移情况等适应征选择亚肺叶切除术式属于手术方式主动选择，根据病变情况制定个体化治疗策略^[23-25]^。目前亚肺叶切除术治疗早期肺癌的优势，以及远期复发与生存期不劣于肺叶切除术的主要证据有力支持亚肺叶切除术治疗早期肺癌的适应症。但是临床实际运用过程中，适应征的执行标准尚不严格，学术界对亚肺叶切除手术方式的选择还存在争议。其中一个重要原因是从胸外科尝试亚肺叶切除术治疗早期NSCLC开始到现在一个相当长的时间里，都是通过影像学资料对早期肺癌进行临床诊断和制定手术策略，只能通过术后病理诊断来反映手术方式选择的正确性。这样的情形不利于主动性适应征个体化术式的准确选择、设计和实施。术中冰冻病理诊断是否能提供准确的早期肺癌分型分期、肿瘤大小、肿瘤与切缘边界，以及淋巴结转移情况来实时精确指导亚肺叶切除术式的选择，从而让患者最大限度获益。最近，国际国内的研究已经开始揭示术中冰冻病理（frozen section, FS）对早期NSCLC亚肺叶切除术个体化治疗的可靠性和指导意义^[18, 23, 26]^。2016年Liu等^[23]^报道的研究对FS诊断早期NSCLC的敏感性和特异性做出评价。803例Ⅰ期周围肺腺癌患者术中FS诊断指导亚肺叶切除术策略。亚肺叶切除手术是否需要扩展为肺叶切除术主要是基于FS。该研究中的早期病变主要分为非典型腺瘤性增生（atypical adenomatous hyperplasia, AAH）、AIS、MIA和浸润性腺癌。术后病理（final pathology, FP）用于评估FS的诊断准确性。FS和FP之间的一致性达到84.4%。在AAH，AIS和MIA的复发低危组，一致率达到95.9%，主要的不一致性在于AIS和MIA病例被FS低估。肿瘤直径≤1 cm和 > 1 cm，FS对肿瘤的诊断准确度分别为79.6%和90.8%；与之相似，2012年Walts等^[26]^的研究中肿瘤直径≤1 cm和 > 1 cm，FS对肿瘤的诊断准确度分别为68%和84.3%。肿瘤 > 1 cm FS诊断更准确的主要原因在于，如果肿瘤小，采样代表性差，容易出现采样误差。肿瘤直径 > 2 cm多为浸润性腺癌，FS多次采样有助于提高诊断准确性。该组病例AIS/MIA，5年无复发生存率为100%，而浸润性腺癌患者为74.1%（*P* < 0.01）。术中FS将使早期周围型肺腺癌患者获得更为精准安全的手术结果，患者恢复更快；基于精准冰冻病理结果的亚肺叶切除术能达到与肺叶切除术相同的治疗效果，复发率低，使手术创伤和治疗效果的平衡更为合理。

## 4 小结

目前多数支持亚肺叶切除术的研究针对Ⅰa期患者，因此术前必须确保分期的准确性，必要时可行PET/CT、纵隔镜或支气管镜超声检查以辅助进行纵隔淋巴结分期，更精准的亚肺叶手术策略应基于术中快速冰冻病理诊断，确定患者手术切除范围。若术中冰冻病理为浸润型腺癌患者，行肺叶切除术和纵隔淋巴结清扫。若病理示良性结节、转移性肺癌，或是不典型腺瘤样增生、原位癌、微浸润腺癌等复发低危患者，则不需要进行肺叶切除。同时肿瘤直径 < 2 cm，肿瘤与切缘距离 > 2 cm或大于肿瘤直径，淋巴结无转移等有利于亚肺叶切除术的重要预后影响因素也可以通过术中冰冻病理确认。对于Ⅰa期复发低危的NSCLC患者，最大程度地保留了其肺功能；对于复发高危的浸润性腺癌患者，肺叶切除则最大程度保证了手术治疗效果。术中冰冻病理结果可以作为一种辅助手段，其诊断结果与最终病理报告符合度非常高，将使早期NSCLC个体化治疗向前迈进重要一步，对于未来早期肺癌选择精准的手术策略具有重要意义^[27]^。目前，相关研究大多是回顾性，更为准确的随机对照前瞻性研究目前正在进行中，待研究结果公布后，关于亚肺叶切除术的选择，我们会有一个更加可信的答案。
